# Comparison of Harrington Rod and Cotrel-Dubousset Devices in Surgical Correction of Adolescent Idiopathic Scoliosis

**DOI:** 10.5812/traumamon.14663

**Published:** 2013-10-14

**Authors:** Ebrahim Ameri, Hasan Ghandhari, Hamid Hesarikia, Hamid Reza Rasouli, Hossein Vahidtari, Navid Nabizadeh

**Affiliations:** 1 Department of Orthopedic Surgery, Shafa Yahyaeian Hospital, Iran University of Medical Sciences, Tehran, IR Iran; 2 Trauma Research Center, Baqiyatallah University of Medical Sciences, Tehran, IR Iran; 3 Department of Orthopedic Surgery, Rasool Akram Hospital, Iran University of Medical Sciences, Tehran, IR Iran

**Keywords:** Harrington, Scoliosis, Surgical Instruments

## Abstract

**Background:**

Since the time of Paul Harrington numerous implants have been introduced for correction of scoliosis, but none are ideal. Newer devices are very expensive, and in our country some patients cannot afford them.

**Objectives:**

The aim of this study was to compare the results of the Harrington rod (HR) device and the newer Cotrel-Dubousset (CD) device in treatment of adolescent idiopathic scoliosis (AIS).

**Materials and Methods:**

A retrospective review assessed patients with AIS admitted for spinal curve correction treated with HR (n = 120) and CD devices (n = 138) between October 1988 to April 2001 at the Shafa Yahyaeian Hospital, Tehran, Iran. We extracted information from the patient’s file and radiographs before, after and two years post-operation.

**Results:**

The mean age of patients was 16.7 ± 2.5 years. There was no statistically significant difference between the two groups regarding gender, age, curve before surgery, and percentage of flexibility. The mean curvature was 70 ± 20.7 in the HR and 64.81 ± 19.4 in the CD group before surgery (P = 0.09); and the mean curvature was 40 ± 16.3 and 26.58 ± 15.37 in HR and CD groups respectively after surgery (P = 0.156). The mean curvature was 47.2 ± 15.9 in HR and 31.2 ± 15.4 in CD groups at two years follow-up (P = 0.156).

**Conclusions:**

Results of many studies have shown no significant impairment in long-term quality of life and function in patients treated with Harrington rods. According to previously performed studies and the current study, surgical correction with Harrington rods seem to be comparable with the newer more expensive CD device. Although there is no doubt that the preference is to use newer devices in view of some disadvantages of HR, but this does not preclude using it for patients that cannot afford the newer devices.

## 1. Background

The term scoliosis, first used by Galen (1, 2), is derived from the Greek word meaning "crooked" (3). Scoliosis has been recognized since Hippocrates. In 1741, Andre devised the crooked spine as his symbol for orthopedics (3). Scoliosis is defined as lateral deviation of the spine greater than 10 degrees on a radiograph. Adolescent idiopathic scoliosis (AIS) is defined as a structural 3-dimensional deformity of the spine, occurring in an otherwise healthy child, for which no recognizable cause exits (4). It is generally acknowledged that the cause of AIS is probably multifactorial (5-7). In addition to the clinical deformity caused by large scoliotic curves, patients with very large curves (> 80°) have increased susceptibility to cardiopulmonary complications, and consequently diminished function and health. Forced expiratory volume in the first second of expiration (FEV1) and forced vital capacity (FVC) decrease linearly with 20% decrease in predicted values with 100° curves or more. Surgical curve correction is aimed to prevent these issues (5, 8). Several studies have been performed regarding the effect of progressive untreated curves by several investigators, including Ponseti and Friedman, Nilsonne and Lundgren, Nachemson, Ascani et al., and Weinstein and Ponseti (9-15). In untreated AIS five problems have been noted: 1) psychosocial effects, 2) back pain, 3) pulmonary dysfunction, 4) curve progression, and 5) mortality (9). These five major considerations are taken into account when deciding to correct the deformity via surgery. Although most experts recommend surgical approach for curves greater than 50 degrees, many other factors should be considered.The goals of surgery for spinal deformity include correction or improvement of deformity, keeping sagittal balance, preservation or improvement of pulmonary function, decreasing morbidity or pain, functional improvement, and improving function or preventing harm of the lumbar spine. To achieve these goals in patients with AIS available surgical methods include anterior, posterior, or combined procedures (4).The ideal fixation device for spinal instrumentation should be safe and reliable, with low failure and breakage. It should be able to resist loads from all directions without external support (brace). Its usage should be easy with minimum operative time. And it should restore normal spinal contours in all planes namely coronal, sagittal, and transverse. Instrumentation should not cause new deformities. The ideal device also should be cost-effective. None of available devices meet all of the ideal criteria (9). Use of distraction rod introduced by Paul Harrington in 1962, combined with posterior arthrodesis and immobilization in a cast or brace for 6 to 9 months has been the standard surgical treatment of AIS for nearly 30 years (9, 16). He initially designed his device for treatment of scoliosis in 129 patients with poliomyelitis. The device consists of ratcheted rods with hooks to obtain and maintain distraction. The hooks are placed in a sublaminar manner at the proximal and distal aspects of the scoliotic deformity (17). To address the limitations of Harrington’s device, Luque developed the concept of segmental sublaminar wires attached to a longitudinal rod. These segmental wires not only increased the stability of the construct, but also provided a greater means of controlling the sagittal contour of the spine. In addition to greater scoliotic correction, it also reduced the need for postoperative immobilization because of the increased stability. Segmental correction and fixation was started by adding sublaminal wiring. Cotrel and Dubousset introduced the concept of using multiple segmental hooks and rods in the late 1970s and early 1980s for correction of scoliosis (18, 19).The expansion of technology since the early 1990s has resulted in the availability of numerous other devices that provide similar advantages (e.g. AO Universal Spine System, Moss Miami instrumentation, Synergy spine system, Cotrel-Dubousset, Horizon, Legacy, Kaneda scoliosis devices and multisegmental transpedicular fixation) (9, 20-22). Harrington implantation has a low (less than 0.5%) incidence of neurologic complications because of minimal invasion of the spinal canal. It provides predictable correction of spinal deformity with little subsequent loss of correction. The Harrington device has a shorter, less difficult "learning curve"; when compared with other newer and more complex devices for instrumentation of spinal deformities. Other preferences of Harrington’s device include: less operating time and blood loss, less hardware, and less cost (23, 24). Incidence of hook dislodgment and pseudoarthrosis in correction of single thoracic curves with Harrington rods are extremely low and near zero (24). Conversely, disadvantages of the Harrington device compared with newer devices include: inability to provide sagittal plane control, limitation of effective derotation of the spine, increase of rib prominence in nearly two-thirds of the patients, higher hook dislodgment and pseudoarthrosis rate in the thoracolumbar and lumbar spine (up to 3 and 4 percent respectively), and necessity for postoperative external support (24). As a result, the Harrington device can be an excellent device for surgical correction of single and double thoracic idiopathic curves in a predictable and safe manner, when limited derotation and sagittal plane control is needed. Also segmental fixation devices appear to be better for fusion of thoracolumbar or lumbar deformities, and those associated with significant sagittal plane deformities (24, 25).

## 2. Objectives

An important drawback of the newer devices is their higher cost. In our country some patients cannot afford the device, because of poverty and lack of sufficient insurance coverage and sometimes surgical correction will have to be delayed. Therefore we may see curve progression and complications. Although there is no doubt in preferences of newer devices, but using cheaper devices does not seem unreasonable in such situations.

## 3. Materials and Methods

A retrospective review was done to assess all patients (n = 258) presenting with idiopathic scoliosis who were treated with Harrington (n = 120) and Cotrel-Dubousset (CD) (n = 138) devices between October 1988 and April 2001 at the ShafaYahyaeian Hospital, Tehran, Iran. The study was approved by our medical research ethics committee. Data were gathered and registered in a data collection form. We extracted general information including age, sex, type of surgery anterior spinal fusion (ASF) and posterior spinal fusion (PSF), and medical history from patient files. Then we measured other parameters including primary curve, curve after the operation, and two years postoperatively from radiographic records, based on the Cobb’s method. We measured and recorded percentage of the flexibility of curve using the bending radiographs. Exclusion criteria were nonidiopathic subjects (congenital, secondary, neuromuscular, etc.), history of spine surgery, cord malformation in MRI or myelography, less than two years follow up, and incomplete radiographs or records. The results were compared between the two groups. 

### 3.1. Statistical Analysis

All data were checked for a normal distribution using the Kolmogorov-Smirnov test. Quantitative variables were compared between groups using the Student’s t-test or Mann-Whitney U-test, depending on whether normal or non-normally distributed variables were used, respectively. For qualitative data, chi-square test and in the absence of circumstances Fisher's exact test was used. Data were analyzed using SPSS 20 software (SPSS, Chicago, IL, USA). A P-value less than 0.05 was considered statistically significant.

## 4. Results

**Figure 1.**
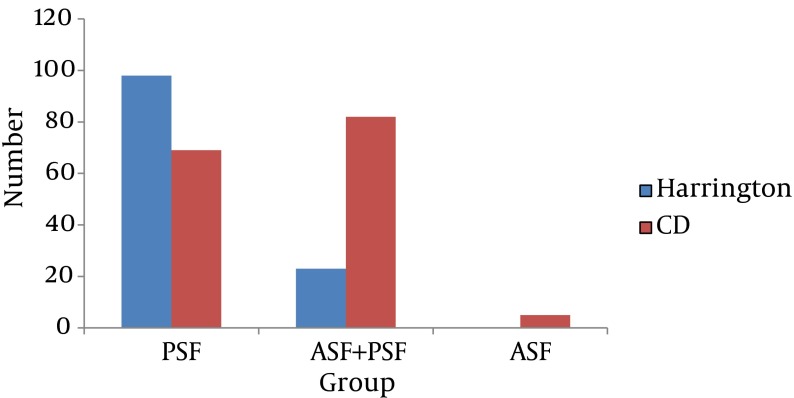
The Mean (ASF, PSF and ASF+ PSF) in the Two Groups Abbreviations: ASF, anterior spinal fusion; PSF, posterior spinal fusion

A total of 258 patients were assessed (120 patients in the HR group and 138 in CD group. The mean age of the patients was 16.7 ± 2.5 years. There was no statistically significant difference between the two groups regarding gender and age (Table 1).The surgical methods (ASF, PSF and ASF + PSF) performed in the two groups are shown in Figure 1. 

**Table 1.** Demographic Data

Items	Harrington	CD ^a^	P value
**Age, Mean ± SD, y**	16.8 ± 2.52	16.57 ± 2.7	0.78
**Gender, Male/Female, No.**	41/79	34/104	0.1

^a^Abbreviation: CD, Cotrel-Dubousset

The mean curve was 70.67 ± 20.63 in all patients before and 33.37 ± 15.9 after the operation (P < 0.01), also the mean flexibility was 38.7 ± 16.6 in all patients. There was no significant difference between the two groups regarding curve before and after surgery and percentage of flexibility (P = 0.09, P = 0.157, and P = 0.686, respectively, Table 2). The mean curve was 39.1±16.1 in all patients at two years follow up after surgery, and there was no significant difference between the two groups (P = 0.12) (Table 2). 

**Table 2.** Mean Curve Before and After Surgery, Mean Curve Two Years After Surgery and Flexibility in Two Groups ^a ^

Items	Harrington	CD ^b^
**Curve before surgery, degree**	70 ± 20.7	64.81 ± 19.4
**Curve after surgery, degree**	40 ± 16.3	26.58 ± 15.37
**Curve after two years, degree**	47.2 ± 15.9	31.2 ± 15.4
**Flexibility, %**	39.67 ± 16.1	37.78 ± 15.5
**Loss of correction, %**	0.18	0.17

^a^No statically significant differences

^b^Abbreviation: CD, Cotrel-Dubousset

## 5. Discussion

In our study curve correction rates were 44% and 58% in HR and CD groups respectively. There was no significant difference between the two groups regarding curve before and after surgery and percentage of flexibility. Loss of correction was slightly higher in the HR group, but not significant. Ideally, when it is accomplished with newer surgical implants, a greater amount of curve correction was achieved. Current devices are able to exert much stronger corrective forces on scoliotic spines than is possible using HD instrumentation. Today, with the introduction of pedicle screw fixation at nearly every level of the spinal segment requiring instrumentation, curve correction is even greater than that achieved with hook-rod segmental fixation devices. These new pedicle screw implant devices, such as CD Legacy, are complex and require a significant amount of training. The established segmental fixation devices-including Cotrel-Dubousset, Texas Scottish Rite Hospital (TSRH), and Isola instrumentation became popular in the mid-1980s and, with refinements which allow the use of more pedicle screw fixation points, remain so today. The device allows the surgeon to achieve increased curve correction, improved sagittal contouring, brace-free postoperative mobilization, and MRI compatibility (with the availability of titanium components) (26). As a testament to its effectiveness, the Luque’s technique is still commonly used for the treatment of neuromuscular scoliosis today (16).The Luque and Harrington devices have been compared, and it was noted that Luque rods maintained sagittal contour better in the thoracolumbar and lumbar spine. Both methods have been found to have similar mean correction (56% with Luque wires *vs.* 55% with HR) (26). A 4.6% failure rate was observed with the Luque technique. One disadvantage of the Luque technique is the passage of wires within the spinal canal with attendant increased risk of neurologic deficit. As the wires are more flexible than hooks, there is an increased risk of inadvertent “plunging” into the canal, which may lead to spinal cord injury. Some studies have observed up to a 17% rate of neurologic deficit with sublaminar wires compared to 1.5% with HR (18). Around the same time that Luque introduced sublaminar wires, Drummond introduced a device of segmental spinous process wiring. The principal advantage of the Drummond technique is that it could be used with rods to achieve curve correction, yet it avoided intrusion into the spinal canal. This device was also used for long fusions, such as neuromuscular scoliosis. The Drummond device combines the use of a Harrington or Luque rod and segmental wire fixation (18). As would be expected, Wisconsin wires were confirmed to have a lower incidence of neurologic deficits relative to Luque wires (8).Initial studies showed varying degrees of success with this technique. In one study, patients had 54% curve correction with only 1.8% loss of correction at early follow-up. In another study, 35 patients were followed for a mean of 6.3 years following the use of Wisconsin wires. Initial curve correction averaged 46% (59° preoperatively to 32° postoperatively). This declined to 29% (Cobb: 36°) at final follow-up 8 years postoperatively (27). The loss of correction was believed to be due to the very posterior vector of control given the placement of wires on the posterior spinous processes. Another disadvantage of Drummond’s device is that it has poor rotational control, and external support is sometimes needed postoperatively (28). A study comparing Cotrel-Dubousset with HR found that CD led to 66.3% correction with 5% loss of correction compared to HR with 51.2% correction and 20.7% loss of correction. Forty percent of those in the HR group developed sagittal imbalance. No cases of sagittal imbalance were seen with CD (29). No neurologic complications were seen in either group. In contrast to the Harrington distraction method, the CD device used various correction methods, including segmental compression and distraction. The CD device also sought to address the rotational component of scoliotic deformity by using rod rotation maneuvers. The amount of derotation achieved with rod rotation maneuvers varied quite a bit across studies. Unfortunately, on balance, the amount of scoliotic derotation achieved with CD appears to be fairly minor (30). In our study curve correction rates were 44% and 58% in HR and CD groups respectively with 7.2% and 4.62% loss of reduction two years after surgery. Amounts of correction in our study are less in both groups comparing to Humke et al., probably because of greater and more rigid primary curves. Some studies were performed to assess long-term result after HR instrumentation for scoliosis correction (23, 30-33). Padua et al. in 2001 evaluated long-term results of HR instrument in 70 patients. Their patients had no important impairment in patient-oriented health related quality of life (34).Cochran et al. assessed 100 cases of AIS correction with HR with 9 years follow up. They compared functional score of patients with controls (without scoliosis), which was equal in both. Although patients with Harrington hooks at L3 and lower had more low back pain, with near equal function and appearances (35). In Bartie s study in 2009 with 19 years follow up of 171 patients treated with Harrington device had just slightly more low back pain compared to controls. And most patients were able to do daily work (23). Gotze in 2002 after evaluation of 82 patients with AIS showed that long term (mean 17 years) results of HR does not negatively influence the quality of life (31). In 2002 Helenius published results of long term function and radiologic evaluation in 87 AIS using HR. Mean values for preoperation, postoperation, and 20 years later curves were 53, 38 and 45 degrees respectively; 22% of patients had degeneration in the lower lumbar spine. And 13% had low back pain. Clinical results and function were not correlated with radiologic results (32). Wojcik compared post-treatment results between HR and CD in AIS, and found that CD had no significant preference in correction of Cobb angle, apical translation and rotation, but was better in T10-T11 translation and rotation above the apical vertebra (36).According to above studies and the current study, surgical correction with Harrington rod seems to be comparable with newer expensive devices. Although, there are some disadvantages related to the older ones, including need for postoperative immobilization, less correction, and possibility of sagittal imbalance, using older and cheaper devices does not appear to be unwarranted in patients who cannot afford the newer ones.
